# Incidence of and risks associated with *Giardia *infections in herds on dairy farms in the New York City Watershed

**DOI:** 10.1186/1751-0147-52-44

**Published:** 2010-06-21

**Authors:** Miguella P Mark-Carew, Yasin Khan, Susan E Wade, Stephanie Schaaf, Hussni O Mohammed

**Affiliations:** 1 Department of Population Medicine and Diagnostic Sciences, College of Veterinary Medicine, Cornell University, Ithaca, NY 14853, USA

## Abstract

**Background:**

The primary aims of this study were to determine the incidence of *Giardia *infections in dairy herds on farms in the New York City Watershed region and to evaluate risk factors associated with infections. Because co-infections of *Giardia *and *Cryptosporidium *spp. are common in this population, we also evaluated the effect of herd infection status on *Giardia *infections.

**Methods:**

Farms were grouped into three cohorts based on their prior infection status with *Giardia *and/or *Cryptosporidium *spp. The sampling plan included collecting fecal samples from all calves below 30 days of age and proportional sampling of calves, young stock, and adults. A total of 10,672 fecal samples were collected and analyzed for the presence of *Giardia *cysts using zinc sulfate flotation. Herds enrolled in the study were sampled seasonally for a study period of two years. The probability of shedding cysts past a certain age and the factors that influenced the likelihood of shedding were evaluated using survival analysis. Linear regression was used to evaluate factors that were associated with the intensity of shedding.

**Results:**

The majority of *Giardia *infections occurred in calves within their first 180 days of age, with the most number of calves shedding *Giardia *cysts between 11 and 20 days of age. The incidence of shedding of *Giardia *cysts ranged from 0.0004 per animal day for cattle in the low risk cohort to 0.0011 per animal day for cattle in the high risk cohort. The likelihood of shedding was influenced by the prior infection status of the herd and the season of collection. Infected animals shed on average 9,658 cysts/gram and the intensity of shedding *Giardia *cysts varied significantly with the age (p < 0.0001) and the season of collection (p = 0.0151 for Spring).

**Conclusion:**

*Giardia *infections are common in dairy herds in the New York City watershed, particularly in calves less than 6 months of age. Seasonality may be an important factor in the perpetuation of infections based on changes in management practices corresponding to weather patterns of a particular season. A dairy herd's prior infection status with *Cryptosporidium *influences the likelihood of infection with *Giardia*.

## Background

*Giardia *is an intestinal flagellated protozoan parasite and has been cited as the most frequent cause of non-bacterial diarrhea in humans. The parasite is a common source of intestinal infections in the developed and developing world with an estimated 2.8 × 10^8 ^cases in humans per year [1,2]. Ingestion of as few as 10 *Giardia *cysts can cause giardiasis [3], and infections are spread via the fecal-oral route by ingestion of cyst-contaminated food or water [4].

The genus *Giardia *has been subject to various taxonomic changes over the years and at one point included over 50 species [2]. As of recent years, there is agreement that six separate species exist: *G. agilis *found in amphibians*, G. ardeae *and *G. psittaci *found in birds*, G. microti *found in muskrats and voles*, G. muris *found in rodents , and *G. duodenalis *found in humans and a wide range of mammalian species [5]. *G. duodenalis *is subdivided into seven assemblages (A-G) that have distinct host preferences (Assemblages C and D for dogs, Assemblage E for livestock, Assemblages F for cats and Assemblage G for domestic rats) [6]. However, Assemblages A and B infect humans and many different species of wildlife, companion animals, and livestock [7].

Research on *G. duodenalis *in livestock, particularly cattle, has shown that the parasite is very common in this population and tends to infect younger calves leading to high prevalence of infection within herds [8,9]. Studies have reported between 45%-73% of calves 0-24 weeks of age having infections [8,10] as well as infection rates as high as 100% [11]. Calves have been reported to be infected with *G. duodenalis *as early as four days of age and have the highest intensity of cyst excretion (10^5^-10^6 ^cysts/gram) between the ages of 4-12 weeks [12]. A study done by our laboratory showed a shedding pattern in dairy calves that increased at 4 days of age and peaked at 14 days [13]. Because dairy cattle can shed high levels of *G. duodenalis *and inhabit watershed areas, there has been much concern about the potential risk of zoonotic *Giardia *infections in human populations.

*G. duodenalis *is etiological agent for diarrheal disease in cattle by itself but is often linked to another common intestinal parasite, *Cryptosporidium *[14,15]. *Cryptosporidium *spp. are similar to *Giardia *in terms of clinical signs, host range, zoonotic potential, and modes of transmission. Many studies have demonstrated concurrent *G. duodenalis *and *Cryptosporidium *spp. infections in dairy calves [16,17] as well as in adult animals [18], yet research on the risk of giardiasis due to co-infection with both parasites has not been fully explored.

In 1993, the Environmental Protection Agency (EPA) concluded that New York City (NYC) met the requirements for filtration avoidance. This meant that filtration was not necessary if the water supply met state and federal raw water standards. The NYC Watershed Agricultural Program, created as part of the filtration avoidance, was established to maintain the quality of the water supply, and the economic viability of agricultural operations in the region. The program focuses on the management of pesticides, sediment, nutrients such as nitrogen and phosphorus, and pathogenic organisms such as *Giardia *and *Cryptosporidium *spp. as they relate to agriculture and water quality [19]. Our study was initiated to obtain information about the epidemiology of *Giardia *and *Cryptosporidium *spp. in dairy cattle as a means of understanding the potential risk of waterborne outbreaks from these protozoa in New York City.

Many prevalence studies on *G. duodenalis *infection in cattle can be found in the literature while only a handful of incidence studies exist. The primary objectives of our study were to determine the incidence of *G. duodenalis *infections in dairy cattle populations in the New York City watershed region and to evaluate the dynamics of *Giardia *infections by taking into consideration factors that may play a role in the perpetuation of infections at the farm level. Because *Giardia *and *Cryptosporidium *are prevalent in dairy cattle operations, we wanted to assess the risk of *Giardia *infections based on the dairy herd's prior infection status of having animals with *Giardia *and *Cryptosporidium*. Finally, we investigated factors associated with the likelihood of infection with *Giardia *and the intensity of cyst-shedding.

## Materials and methods

We carried out a longitudinal follow-up study to address the stated objectives. The target population consisted of cattle on dairy farms in the Catskill/Delaware Watershed of New York City. The watershed is located in southeastern New York State and houses approximately 200 dairy operations located within the catchment area. The study population was drawn from herds enrolled in a voluntary program administered by the Watershed Agricultural Council (WAC). Approximately half of the herds in the target population were surveyed for the prevalence of *Giardia *in a cross-sectional study [19]. A total of 40 herds were selected from that study population for enrollment in this study based on their willingness to participate.

To account for the initial infection status of the population, study farms were classified into one of three protozoan risk levels based on prior herd infection status [19]. High-risk farms were defined as those on which both *Giardia *cysts and *Cryptosporidium *oocysts were detected (eight farms). Farms on which only *Giardia *was detected were classified as the intermediate risk group (twenty-five farms). Farms included in the lowest risk category were those on which neither *Giardia *cysts nor *Cryptosporidium *oocysts were previously detected (seven farms).

Animals in these herds were randomly sampled using an age stratified sampling design. Three strata of age groups were created: ≤6 months of age (calves), 6 to 24 months of age (young stock), and > 24 months of age (adults) [19]. All farms were visited on a seasonal basis (three times a year) for two years starting in June of 1995 and ending in June 1997. Newborn animals, perceived as high risk for infection, were sampled within a week of birth once the decision was made to keep them in the herds. Calves that were negative for *Giardia *infections at the first sampling were sampled again at subsequent farm visits.

### Sample Collection and Analysis

Fecal samples were collected rectally from each animal, immediately placed in uniquely labeled specimen containers and stored on frozen cold packs until transported to the laboratory. Once at the laboratory, samples were stored at 4°C. For each sample, three grams of feces were processed using zinc sulfate (sg 1.18) as the flotation medium [20]. *Giardia *cysts were enumerated by trained counters using bright field microscopy at 200× magnification. An animal was considered *Giardia-*positive if at least one *Giardia *cyst was detected with the correct morphology (i.e. optical properties, internal structure, size and shape). For samples with less than 100 cysts/gram, the entire cover slip was counted. For samples with high cyst numbers, 20 random fields were counted and the estimated cyst count/gram was determined by multiplying by the number of fields on the cover slip. All identification techniques were done at the Cornell University Animal Health and Diagnostic Center (Ithaca, NY).

### Data collection

Data on intrinsic factors (age, breed, and sex) related to a specific animal were collected by personal interview of the farm owner and examination of herd records. The date of collection was also recorded to adjust for the risk of shedding by season and to determine animal ages.

### Statistical Analysis

In the data analyses, the sampling periods were grouped into three seasons representing months with similar weather patterns: winter (November through March), spring (April through June), and summer (July through October). The probability that an animal would shed *Giardia *cysts past a specific time was computed using the survival analysis approach (Statistix 8.0). Kaplan Meier survival curves were used to present the pattern of shedding in relation to the age of the animals, and significant differences between the three risk groups of animals were evaluated using the Log-rank test [21]. The Cox proportional hazard model was used to evaluate the likelihood of shedding in a short time period given the season and prior status of the herd [22]. Because the sampling units in this study, the cattle, are clustered in herds, it was assumed that this clustering would lead to a correlation in the likelihood of infection within the study population. This correlation between responses occurs because they are dependent on exogenous factors that are associated with these responses (i.e. infection with *Giardia*). Conditioning on an observed set of these factors by controlling for their effect in the analysis and including them as covariates in the logistic regression analysis will sometimes achieve approximate conditional independence. However, more often this correlation in the response arises from both observed and unobserved risk factors. It was assumed that the unobserved risk factors were randomly distributed among farms and the overall significance of this assumption was evaluated by using a mixed-effect logistic regression model [23] using Egret software (Cytel Statistical Software, MA).

Regression analysis, with an appropriate log transformation for the number of cysts shed, was employed to identify factors associated with the intensity of shedding of *Giardia *cysts given the age of the animal, the prior risk group, and season of sampling. The significance of a factor was evaluated by the significance of the respective regression coefficient (p-value < 0.05). The analysis was performed using SAS 9.2 (SAS Statistical Software, Raleigh, NC).

## Results

A total of 10,672 fecal samples were collected over the course of the study. For statistical analyses, data from 4,938 unique dairy animals from ages 1 day to 730 days of age were used. We diagnosed 1,236 animals as shedding *Giardia *cysts during the study period. The cumulative incidence of *Giardia*, computed as the proportion of new cases of *Giardia *over the course of the two-year study, was 25% (1,236/4,938). Animals in low risk herds had the lowest incidence rate (0.0004 per animal day = 96 new cases/243,501 days) in comparison to animals from moderate risk (0.0006 per animal day = 603 new cases/975,890 days) or high risk herds (0.0011 per animal day = 537 new cases/501,710 days).

**Figure 1:**
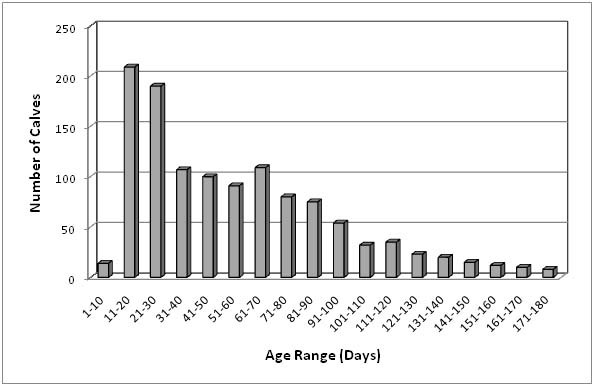
**Frequency distribution of the age of animals, up to 180 days of age, among young stock diagnosed as shedding *Giardia *during the study period**. The range is measured in ten-day intervals.

Among all animals that were diagnosed as shedding *Giardia *cysts, 95.8% were calves (1,184/1,236). The earliest age at which a calf shed *Giardia *was 2 days. Figure 1 shows the frequency distribution of the age of young animals up to 6 months of age (≤ 180 days) that were diagnosed as shedding *Giardia *cysts among the study population. The most number of calves began shedding *Giardia *between 11 and 20 days of age (220 animals). The average number of estimated cysts shed was 9,658 cysts/gram with a range from 1 cyst/gram to 1.75 × 10^6 ^cysts/gram.

**Figure 2:**
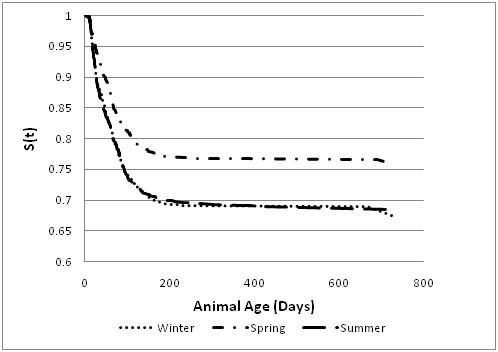
**Kaplan-Meier survivorship curve for the probability of not shedding *Giardia *passed certain age for animals enrolled in the study and grouped by season of the year**. The data was restricted to a follow-up period of 730 days.

A total of 428 new cases of *Giardia *were diagnosed in the summer, 452 cases in the winter, and 356 cases in the spring. Winter had the highest incidence rate of shedding (0.00083 per animal day) while spring had the lowest rate (0.00057 per animal day). There were no significant differences between crude incidences among the three seasons. Figure 2 shows the plot of the survivorship function for the likelihood of not shedding *Giardia *cysts over time based on season of sampling. The analysis was limited to animals that were two years of age or less. The probability of shedding *Giardia *past a certain age was higher in winter and summer in comparison to spring (p < 0.05) (the survival curve for spring is higher than that for winter or summer). There was no significant difference between winter and summer. We examined the potential likelihood of clustering of shedding of *Giardia *cysts by farm using random effect models. There was no evidence of hierarchal clustering and the likelihood of shedding *Giardia *cysts was randomly distributed by farm (data not shown).

The probability of an animal shedding *Giardia *cysts past a certain age was evaluated among the three risk cohorts using survival analysis (data not shown). The analysis was again restricted to a follow up period of up to 730 days. All animals had high incidence of shedding *Giardia *cysts earlier in their lives. Ten percent of the animals from farms in the low risk cohort were found to shed cysts within 49 days of age while an equivalent percentage in the moderate and high risk groups become infected within 35 and 27 days, respectively (the 90% percentile of the survival function). There was a significant difference in the risk of shedding among the three groups (p < 0.05). The probability of shedding *Giardia *cysts within the first year for the low, moderate, and high risk groups was 21, 31, and 45%, respectively. There was no evidence that there was a clustering of incidence of *Giardia *cyst-shedding by farm beyond chance alone as was evaluated with the mixed effect models analysis (data not shown).

The results of investigating the likelihood of shedding *Giardia *cysts in a specific season while controlling for the prior herd risk are shown in Table 1. Animals sampled in spring were less likely to be diagnosed as shedding *Giardia *cysts compared to animals that were sampled in winter (hazard ratio = 0.7). The risk of shedding *Giardia *cysts in animals that were sampled in summer was not significantly different than the risk for animals that were sampled in winter when we adjusted for the herd status prior to enrolling animals. Animals that were sampled from moderate or high risk farms had an increased likelihood of shedding *Giardia *cysts in comparison to animals that were from low risk herds.

**Table 1:** The impact of season of collection and the prior status of the herd on the hazard of shedding *Giardia *cysts among dairy cattle in the study population.

Risk factors	Regression coefficient	Standard error	Hazard ratio	95% CI
Prior risk of *Giardia*				
*Low risk*	0		1.0	
*Moderate risk*	0.328	0.109	1.4	1.1, 1.7
*High risk*	0.620	0.110	1.9	1.5, 2.3
Season of the year				
*Summer*	0		1.0	
*Winter*	0..058	0.080	1.1	0.9, 1.2
*Spring*	-0.266	0.083	0.7	0.6, 0.9

Low risk= Herds shedding neither *Giardia *or *Cryptosporidium *prior to start of the study

Intermediate risk= Herd shedding *Giardia *only prior to the start of the study

High risk= Herd shedding both *Giardia *and *Cryptosporidium *prior to the star of the study

Winter= November through March

Spring= April through June

Summer= July through October

**Figure 3:**
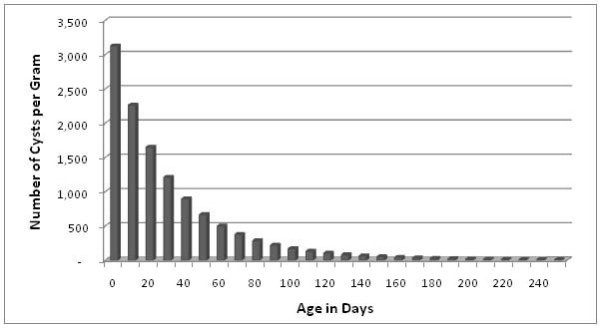
**The pattern of intensity of shedding *Giardia *cysts by age of the animal for all animals enrolled in the study and diagnosed as shedding cysts during the follow-up period (730 days)**.

Risk factors that were hypothesized to be associated with the intensity of shedding *Giardia *cysts were evaluated (Table 2). The intensity of shedding *Giardia *cysts decreased significantly with the age of the animal (Figure 3). The figure is produced by substituting the age of the animal into the equation: Y= 3.4949 - 0.0142age + 0.000015age^2 ^(where Y = log of number of cysts shed/gram of feces). Younger animals were shown to shed higher numbers of cysts and the pattern of shedding decreases with age reaching its minimum value around 250 days. The shedding intensity also varied by the season of the year. Animals sampled in winter and spring shed fewer cysts per gram in comparison to animals sampled in summer (as seen by negative regression coefficients for the former) (Table 2).

**Table 2:** Factors that affect the intensity of shedding *Giardia *in dairy herds in the New York City Watershed.

Risk Factor	Regression Coefficient	Standard Error	Pr > |t|
*Age*	-0.0142	0.000980	<0.0001
*Age*^2^	0.000015	1.367E^-6^	<0.0001
Prior Infection Status			
*High risk*	0		
*Low Risk*	0.2201	0.0840	0.0089
*Intermediate Risk*	-0.0288	0.0539	0.5939
Season of Infection			
Summer	0		
*Winter*	-0.0554	0.0464	0.2324
*Spring*	-0.1205	0.0495	0.0151
Constant	3.4949	0.0684	<0.0001

Low risk= Herds shedding neither *Giardia *or *Cryptosporidium *prior to start of the study

Intermediate risk= Herd shedding *Giardia *only prior to the start of the study

High risk= Herd shedding both *Giardia *and *Cryptosporidium *prior to the star of the study

Winter= November through March

Spring= April through June

Summer= July through October

## Discussion

Several studies have reported on the prevalence of *Giardia *spp. on dairy farms using a cross-sectional study design [12,24]. Our study differs from these because we used a longitudinal approach in which the occurrence of the protozoa among dairy herds in the watershed was observed over a time period of two years. The approach we undertook, by virtue of its design of repeated sampling, shed more light on the dynamic of the infection in the population to a cross-sectional approach which reflects a one-time sampling [22].

Because previous studies have shown associations between *Giardia *and *Cryptosporidium *infections, we assessed the incidence of *Giardia *in dairy herds with a prior history of co-infection with both parasites as compared to herds with a prior history of infections with only *Giardia *and herds without infections with either parasite. Animals in the high risk group had the greatest chance of shedding *Giardia *cysts within their first year in comparison to the low and moderate risk groups. Our analyses showed that the high risk group had the least number of animals that remained *Giardia-*-free over the course of two years. It is likely that combined *Giardia *and *Cryptosporidium *infections impacted the probability of shedding and potential for the spread of infection in this group. It is important to note that the number of farms in each risk group was not equal and that the high risk group included the least number of farms. We cannot rule out the possible effect of this on our analyses.

Seasonality was shown to play a significant role in the probability of dairy cattle becoming infected with *Giardia*, particularly during the winter and summer months. Our results are in line with previously published studies that show season being associated with risk of infection in dairy cattle [19,25]. Seasonal weather patterns influence management practices of dairy farms in the New York City Watershed region in that animals are confined in barns during the winter months and are allowed to roam in pastures during the summer. We speculate that being in close quarters during the winter and having access to streams and grazing land that may be contaminated with *Giardia *cysts from wildlife, other cattle within the herd, and human waste during the summer months may serve to propagate infections during these two seasons. Furthermore, we believe that the probability of infections as well as incidence rates were lowest in the spring because the calving season in this area begins in late summer and continues through October, meaning that young animals were less prevalent in herds.

Results are in agreement with previous studies showing that calves start shedding *Giardia *cysts shortly after birth [24]. Calves began shedding *Giardia *by 2 days of age, three days earlier than we had previously reported [26]. Only 4% of dairy cattle that shed *Giardia *over the course of the study were over 6 months of age.

Age, coming from a herd with a prior history of shedding *Giardia *cysts, and shedding *Giardia *in spring were determined to be risk factors associated with the intensity of shedding *Giardia *cysts. Evidence in support of age being a risk factor for infection is common throughout the literature [19,27,28]. Based on microscopic analysis, cysts counts from calves shedding *Giardia *varied from one cyst/gram to as high as 1,759,824 cysts/gram. The animal that shed over a million cysts was 28 days old. The mean number of cysts/gram shed by all cows in the study (including young stock and adults) was 9,658 cysts/gram and by calves only was 10,069 cysts/gram. Our estimates are much higher than other published reports [10,13,29] and may be attributed to high prevalence of *Giardia duodenalis *in the New York City Watershed ecosystem.

Calves begin shedding both *Giardia *and *Cryptospordium *early in their development and can spread infections to other animals within the herd. Additionally, depending on the strain of *G. duodenalis *being shed, there is potential for infections to spread to other animal populations, including humans. The spread of the infection within the farm would contribute to an increase in the herd level of endemicity. Our analyses support this since herds in the moderate and high risk cohorts were more likely to have infections than herds without prior history of infection (Table 1).

This study provides information regarding the incidence of *Giardia *in cattle in watersheds and the dynamics of the infection as they relate to the endemicity of the protozoan in these populations. We believe that *Giardia *and *Cryptosporidium *are epidemiologically linked, based on their common hosts, mode of transmission and similar clinical manifestations. Though levels of endemicity differ for the two intestinal parasites (*Giardia *infections often being cited as more prevalent in dairy cattle than *Cryptosporidium *infections) [9,17,30], we argue that efforts to control *Giardia *infections in dairy cattle herds in the New York City Watershed should target both protozoa.

Recent studies have shifted from using zinc sulfate flotation to more advanced diagnostic tests such as immunoflurorescence antibody test (IFAT) and enzyme-linked immunosorbent assay (ELISA) [29,31].. We feel confident that our flotation technique was sufficient for identifying and enumerating *Giardia *cysts, though others have reported that the sensitivity of the test is substantially lower than other techniques [31]. We cannot rule out the possibility that our incidence values are underestimated.

Given that this study was a follow-up to a previously published study where we sought to identify risk factors associated with *Giardia *infections in the target population of dairy cattle in the New York City Watershed, we did not include herd management factors in our analysis [19]. We believe that we accomplished our goals without including these factors but recognize that their addition in our analyses would have strengthened our results.

Future directions point to molecular analyses of fecal samples collected in this study in order to identify assemblages of *G. duodenalis *and to assess the potential for zoonotic transmission of *G. duodenalis*. We understand that the age of the samples may be a hindrance to obtaining this information, but some studies have shown successful PCR amplification of samples as old as ours [32]. Published reports have found Assemblages A, B, and E in dairy cattle worldwide [8,23,33]. We plan to contribute to the literature novel molecular information on the types of *G. duodenalis *infections found in dairy herds in the New York City Watershed through PCR analysis of the triosephosphate isomerase (*tpi*) and beta-giardia (*bg*) genes and subsequent sequencing to identify both zoonotic and livestock-specific strains in this population.

## Competing interests

The authors declare that they have no competing interests.

## Authors' contributions

MPM performed data and statistical analyses, interpreted results and drafted the manuscript. YK conducted statistical analyses. SEW assisted in designing the study and participated in the laboratory work. SS assisted in designing the study and participated in the laboratory work. HOM conceived, designed supervised and coordinate different activities including data analysis. All the authors red and approved the final manuscript.
